# ROS-Activated Ion Channels in Plants: Biophysical Characteristics, Physiological Functions and Molecular Nature

**DOI:** 10.3390/ijms19041263

**Published:** 2018-04-23

**Authors:** Vadim Demidchik

**Affiliations:** 1 Department of Horticulture, School of Food Science and Engineering, Foshan University, Foshan 528000, China; dzemidchyk@bsu.by; Tel.: +375-172095934; 2 Department of Plant Cell Biology and Bioengineering, Biological Faculty, Belarusian State University, 4 Independence Avenue, 220030 Minsk, Belarus; 3 Russian Academy of Sciences, Komarov Botanical Institute, 2 Professora Popova Street, 197376 St. Petersburg, Russia

**Keywords:** reactive oxygen species, ROS, ion channels, calcium signaling, potassium ions, electrolyte leakage, copper ions, hydroxyl radicals, plant growth regulation, plant stress physiology

## Abstract

Ion channels activated by reactive oxygen species (ROS) have been found in the plasma membrane of charophyte *Nitella flixilis*, dicotyledon *Arabidopsis thaliana*, *Pyrus pyrifolia* and *Pisum sativum*, and the monocotyledon *Lilium longiflorum*. Their activities have been reported in charophyte giant internodes, root trichoblasts and atrichoblasts, pollen tubes, and guard cells. Hydrogen peroxide and hydroxyl radicals are major activating species for these channels. Plant ROS-activated ion channels include inwardly-rectifying, outwardly-rectifying, and voltage-independent groups. The inwardly-rectifying ROS-activated ion channels mediate Ca^2+^-influx for growth and development in roots and pollen tubes. The outwardly-rectifying group facilitates K^+^ efflux for the regulation of osmotic pressure in guard cells, induction of programmed cell death, and autophagy in roots. The voltage-independent group mediates both Ca^2+^ influx and K^+^ efflux. Most studies suggest that ROS-activated channels are non-selective cation channels. Single-channel studies revealed activation of 14.5-pS Ca^2+^ influx and 16-pS K^+^ efflux unitary conductances in response to ROS. The molecular nature of ROS-activated Ca^2+^ influx channels remains poorly understood, although annexins and cyclic nucleotide-gated channels have been proposed for this role. The ROS-activated K^+^ channels have recently been identified as products of Stellar K^+^ Outward Rectifier (*SKOR*) and Guard cell Outwardly Rectifying K^+^ channel (*GORK*) genes.

## 1. Introduction

Ion channels are proteinaceous pores in membranes that play indispensable roles in plant physiology. Originally, they were explored as systems for uptake of minerals in ionic form for nutritional and osmotic needs and generation of the electric potential difference across the membrane [1]. Over the last two to three decades, ion channels have been shown to be involved in much more than initially expected [2,3,4,5]. New functions included elongation growth of root cells [6] and pollen tube [7], regulation of programmed cell death [8], sophisticated stomatal behavior [9,10,11], control of stress-induced signaling and electrolyte leakage [12,13,14], and even the regulation of photosynthesis [15,16]. One of the recent findings is that ion channels are involved in redox-dependent processes and are capable of sensing reactive oxygen species (ROS), which are abundantly synthesized by plant cells during expansive growth, and response to hormones and stresses [10,12,13,17,18,19,20,21].

ROS-activated ionic conductances in plant membranes were originally reported in relation to Cu^2+^ toxicity in the charophyte algae *Nitella flexilis* by Demidchik et al. [17,22]. We now know that their functions are much broader. Modern schemes of plant regulatory networks, such as the action of signaling molecules, stress perception, and stimulation of elongation growth have included the ROS-activated channels as key components [23,24]. The interest in these systems is in linking transmembrane fluxes of Ca^+^, K^+^ and other ions to the production of ROS. Both phenomena are ubiquitous and crucial for plant life. Acting in concert, ROS-generating, and Ca^2+^- and K^+^-transporting systems can control most physiological and pathophysiological reactions. The purpose of this review is to summarize available data on ROS-activated channels in plants with an emphasis on their biophysical properties, physiological functions, and genetic background.

## 2. First Observations of ROS-Activated Ion Fluxes: Electrolyte and K^+^ Leakage in Response to Stresses

The first evidence for stimulation of ion fluxes in plant tissues treated with ROS was obtained from measurements of so-called electrolyte leakage caused by a transition metal copper ions—Cu^+/2+^, which is a free radical capable of donating and accepting electrons from hydrogen peroxide (H_2_O_2_), superoxide anion radicals (O_2_^•−^), nitric oxide (^•^NO) and other ROS and reactive nitrogen species (RNS) [14,25]. Cu^+^ reduces oxygen in H_2_O_2_ molecule to extremely reactive hydroxyl radical (HO^•^), in the Haber-Weiss cycle based on Fenton-like reactions [25,26,27,28,29]. McBrien and Hassall [30] treated *Chlorella vulgaris* cells with copper ions and found a dramatic loss of K^+^ from these cells, accounting for 94% of all cell K^+^ at approximately 0.3 mM CuSO_4_. Detectable K^+^ activities in medium were measured in the few minutes after the addition of copper ions. Very similar rapid K^+^ release was found in tests on two species of marine algae, *Dunaliella tertiolecta* and *Phaeodactylum tricornutum* treated with copper ions [31]. Studying the sensitivity of *Chlorella* cells to heavy metal ions and oxidants, De Filippis [32] showed that not only Cu^2+^, but also Hg^2+^, as well as methylmercuric chloride and phenylmercuric acetate, can induce K^+^ efflux [32]. All these agents are redox-active and can produce ROS.

The first evidence for ROS-induced ion fluxes in higher plants was probably obtained in the study of electrolyte leakage from Cu^2+^-treated *Agrostis tenuis* plants [33]. In these experiments, root growth inhibition and K^+^ efflux were measured and compared in Cu^2+^- and Zn^2+^-treated *Agrostis tenuis* populations with different sensitivities to these heavy metal ions. Experiments demonstrated that Zn^2+^ was without effect on the release of K^+^ from the root tips, but all the concentrations of Cu^2+^ tested (0.01–1 mM) caused dramatic efflux of K^+^, although the effect was less in the case of the Cu-tolerant populations than for the others. Similar results were obtained for Cu^2+^-treated *Silene cucubalus* roots [34] and *Avena sativa* leaves [35]. These tests were additionally accompanied by treatment with SH-oxidising and ROS-generating reagents, such as *N*-ethylmaleimide, cumene hydroperoxide and *p*-chloromercuribenzoate, which all caused effects similar to Cu^2+^, demonstrating that Cu^2+^-induced ROS, but not the specific characteristics of copper, are the reason of K^+^ efflux from cells. An involvement of potassium channels in K^+^ release was finally demonstrated by Murphy and Taiz [36] and Murphy et al. [37] working on *Arabidopsis thaliana* varieties with different copper sensitivity. They found that K^+^ loss from *Arabidopsis thaliana* roots is a good indicator of general sensitivity of plants to Cu^2+^, and that it is inhibited by a specific K^+^ channel blocker—tetraethylammonium ions (TEA^+^). Further studies of K^+^ efflux from *Arabidopsis thaliana* root demonstrated that Cu^2+^ ions can induce hydroxyl radical production in intact roots which increases upon addition of H_2_O_2_ and ascorbate [12]. Using Microelectrode Ion Flux Estimation™ (MIFE™) technique, Demidchik et al. [12,13] showed that the hydroxyl radical-generating mixture containing Cu^2+^, H_2_O_2_, and ascorbate causes massive and reproducible TEA^+^-sensitive K^+^ efflux from *Arabidopsis*, pea, corn, spinach, wheat, and clover roots, which was lost in the *Arabidopsis* K^+^ channel knockout mutant *Atgork1-1*, lacking K^+^ efflux channel AtGORK (*Arapidopsis thaliana* Guard cell Outwardly Rectifying K^+^ channel). 

ROS generation is ubiquitous in most stresses, such as salinity, freezing, pathogen attack, drought, heavy metals, hypoxia, ultraviolet, ozone, and many others [29]. Intriguingly, cation channel-mediated electrolyte leakage always accompanies these stresses [14]. It can be speculated that K^+^ leakage during stress response is induced by ROS (in addition to commonly observed depolarisation). The idea of an ion channel mechanism of stress-induced electrolyte/K^+^ efflux was proposed by Palta et al. (1977) [38], based on studies of freezing tolerance, which has now been shown to be dependent on ROS generation [39]. Atkinson et al. (1985, 1990, 1996) found the sensitivity of pathogen-induced K^+^ efflux to cation channel blockers, such as La^3+^, Gd^3+^, and Co^2+^, and concluded that there was an involvement of cation channels [40,41,42]. It is becoming clear that stresses caused by pathogens rely on de novo ROS synthesis and sometimes severe oxidative stress [43]. Electrolyte and K^+^ leakage induced by high levels of NaCl was observed in the 1960s [44]. Originally, this effect was attributed to the non-specific membrane damage. However, in the 1970s, Nassery (1975, 1979) provided evidence that high levels of NaCl specifically induces efflux of K^+^, but not other ions. Analyzing K^+^ efflux from wheat, barley, bean, and chick pea roots, Nassery (1975, 1979) demonstrated that this reaction was sensitive to K^+^ channel blockers and that it was not induced by osmotic stress [45,46]. Similar reactions have been found in drought- and heat-treated plants, which demonstrated dramatic electrolyte/K^+^ efflux [47,48]. Recent findings showed that NaCl can trigger production of hydroxyl radicals in roots of higher plants, which stimulate K^+^ efflux via K^+^ channel GORK activation [13]. 

In parallel to K^+^ release studies, Price [49] and Price et al. [50], using *Commelina communis* leaf epidermal strips and aequorin-transformed *Nicotiana plumbaginirolia* seedlings, demonstrated that exogenously applied ROS or ROS-generating chemicals (0.05–1 mM H_2_O_2_ and 0.1 mM paraquat) can induce influx of Ca^2+^ from extracellular space to the cytosol transiently increasing cytosolic free Ca^2+^. Effects were inhibited by verapamil, a Ca^2+^ channel antagonist, which is widely used in animal ion channel studies, pointing to involvement of ROS-activated Ca^2+^-permeable ion channels. Clayton et al. [51] demonstrated very similar elevation of cytosolic Ca^2+^ in response to high levels of O_3_, which is a critically important abiotic stress factor and, at the same time, it is a ROS. This increase in cytosolic Ca^2+^ was inhibited by La^3+^, which is a non-specific inhibitor of cation channels in plants. Demidchik et al. [12,18], utilising Ca^2+^-aequorin luminometry and MIFE techniques, found that HO^•^-generating mixtures (Cu^2+^, H_2_O_2_ and ascorbate) induced Ca^2+^ influx, which was sensitive to the range of cation channel blockers and antioxidants. 

## 3. Cu^2+^-Activated Non-Selective Cation Conductances in Charophyte Algae

Following the observations of Cu^2+^-induced ion leakage from alga cells [30,31,32], Demidchik et al. [17,22] examined effects of Cu^2+^ on major types of ionic currents in charophyte *Nitella flexilis* and discovered Cu^2+^-activated cation conductances, which were rapidly activated after the addition of Cu^2+^ to the bathing solution. This microelectrode voltage-clamp study had an advantage in the preservation of the cell wall, where H_2_O_2_ and ascorbate can catalyse HO^•^ generation in the presence of Cu^2+^ [29], with minimal cell damage since it was impaled just by one microelectrode (compared to patch-clamp techniques). Demidchik et al. [17,22,52] showed that exposure of intact *Nitella flexilis* cells to redox-active transition metals, Cu^2+^ and Fe^3+^ (5–100 µM), induced voltage-independent rapidly activating conductance, which showed permeation of different cations, but did not allow passage of anions [17,22,52]. This conductance was blocked by nifedipine (Ca^2+^ channel blocker) and had temperature coefficient Q_10_ between 1.2 and 1.6, suggesting the involvement of an ion channel-based mechanism rather that of active transporters, which have higher Q_10_. Cu^2+^-activated cation conductance in *Nitella flexilis* explained the mechanism of high heavy metal toxicity in green alga. Increase in non-specific cation efflux caused by Cu^2+^ resulted in irreversible collapse of the electric potential across the plasma membrane and stable loss of cell turgor. Thus alga cells died due to activation of the cation channels by very low concentrations of Cu^2+^ (10–30 µM). The effect of cell death was prevented by addition to the bathing solution of cation channels blockers within 20–30 min after Cu^2+^ application. These studies also demonstrated that Cu^2+^ blocked *Nitella* anion channels and H^+^-ATPase, demonstrating specificity of the effect on the non-selective conductance [17,22].

## 4. Ca^2+^ Influx Activated by H_2_O_2_ in Leaves

The first report on ROS-activated cation channel in leaves of higher plants was published in 2000 by Pei et al. [10] who studied Ca^2+^-permeable channels in guard cells in relation to the mechanism of stomata closure. These authors have found that exogenously applied H_2_O_2_, which was probably produced in response to abscisic acid, activated Ca^2+^-permeable non-selective cation channels in the guard cell plasma membrane. The channels were sensitive to phosphorylation status [53]. In *abi2-1* protein phosphatase mutants, the H_2_O_2_-mediated activation of guard cell Ca^2+^-permeable channels was suppressed and was insensitive to abscisic acid [24]. Further biochemical analyses of phosphatase 2C (PP2C) showed that this protein is directly inhibited by H_2_O_2_ and could be one of the prime targets for H_2_O_2_ in guard cells [54]. Another possible scenario is that PP2C dephosphorylates some intermediate regulators controlling gating of ROS-activated Ca^2+^-permeable channels [55]. Moreover, the phosphatase can block NADPH oxidase activation and ROS production evoked by abscisic acid, resulting in no ROS generation and no channel activation [24].

The function of ROS-activated Ca^2+^-permeable channels in guard cell plasma membrane relates to the need for decreasing the turgor pressure when stomata close under drought stress. Ca^2+^ influx increases the cytosolic Ca^2+^, which activates anion channels, efflux of anions and water loss from guard cells needed for stomatal closure [53]. The guard cell ROS-activated Ca^2+^-permeable channels are activated by NADPH oxidase-generated ROS while the NADPH oxidase, in turn, is stimulated by the drought stress phytohormone, abscisic acid (reviewed by Murata et al. [24]). Moreover, salicylic acid can also participate in this reaction but via peroxidase-dependent ROS production, resulting in very similar Ca^2+^ influx-mediated phenomena (reviewed by Sierla et al. [56]). Intriguingly, Wu et al. [57] have recently shown that leaf mesophyll ROS production for activation of K^+^ efflux can be catalyzed by disruption of electron flow in the chloroplast electron transport chains. This suggests a different pattern of regulation comparing to the guard cells.

## 5. ROS-Activated Cation Channels in Roots

In 2003, Demidchik et al. [12] and Foreman et al. [6] provided the first evidence that ROS-activated cation channels exist in roots of higher plants. The idea that, similar to charophytes, Cu^2+^ and hydroxyl radicals can activate ionic conductances in the plasma membrane of higher plants was tested using *Arabidopsis thaliana* roots [6,12,13,18,58]. The protoplasts, which are used in patch-clamp studies, lack apoplastic H_2_O_2_ and ascorbate (because they do not contain cell walls), which can be required for hydroxyl radical production by Cu^2+^ [59]. Therefore, Cu^2+^ was applied to root epidermal protoplasts together with ascorbate (Cu/Asc) or as three-component mixture of Cu^2+^, ascorbate and H_2_O_2_ (Cu/Asc/H_2_O_2_). These mixtures are used by animal physiologists studying ROS-activated conductances [60]. Demidchik et al. [12] demonstrated that Cu/Asc activated two types of conductances in protoplasts isolated from root mature epidermis, including Ca^2+^-permeable inwardly-directed and K^+^-selective outwardly-directed conductances, respectively. The Ca^2+^-permeable conductance was voltage-independent and non-selective, showing the following permeability series: K^+^ (1.00) ≈ NH_4_^+^ (0.91) ≈ Na^+^ (0.71) ≈ Cs^+^ (0.67) > Ba^2+^ (0.32) ≈ Ca^2+^ (0.24) > TEA^+^ (0.09). The K^+^ efflux conductance demonstrated typical Shaker-like outward rectification with relatively high selectivity for K^+^: K^+^ (1.00) > Na^+^ (0.31) >> Ba^2+^ (0.06) >TEA^+^ (0.05). These selectivity series were typical for *Arabidopsis thaliana* ‘K^+^ outward rectifiers’ (KOR) reported by [61]. Surprisingly, the kinetics of activation over time for *Arabidopsis* cation currents after Cu/Asc addition was similar to the time-dependence of Cu^2+^-induced conductances in *Nitella*, suggesting the involvement of a similar activation mechanism. Interestingly, Cu^2+^ added without ascorbate did not activate conductances in protoplasts [6,12] although it caused elevation of cytosolic free Ca^2+^ in intact roots [12] and induced currents in intact *Nitella* cells [17]. This indicates that apoplastic ascorbate and H_2_O_2_ are probably required for HO^•^ generation via the Haber-Weiss cycle in the cell wall. Hydrogen peroxide for these reactions is likely to be produced by NADPH oxidases because, when cell wall peroxidases were removed by the protoplast isolation procedure, Cu/Asc was still capable of activating currents [6]. Moreover, knockout mutants lacking NADPH oxidase RBOHC, were deficient in Ca^2+^ flux needed for root cell elongation [6]. Peroxidase and oxidase sources of ROS in the apoplastic space can be important during stress responses, but the evidence that this source is involved in activation of ion channels is still missing [29].

Based on the higher ROS-activated Ca^2+^ conductance in growing cells compared to mature non-growing cells, and inhibition of root hair growth by cation channel antagonists, a mechanism of cell expansive and polar growth, has been proposed by [6]. According to this mechanism, the localised elevation of ROS-activated Ca^2+^ influx in growing cell parts leads to up to 10 times higher Ca^2+^ activity in the cytosol, which stimulates exocytosis and the delivery of new cell structural material [6,62,63,64]. NADPH oxidase provides most ROS for this Ca^2+^ loading [6,62]. This mechanism has been confirmed in other species, for example in young roots of *Mesembryanthemum crystallinum* [65] and *Salix nigra* [66]. ROS-activated Ca^2+^ influx in growing root cells is up-regulated by auxin (a major hormone inducing root growth) via stimulation of expression of NADPH oxidases and peroxidases by transcription factor RSL4, which in turn is transcriptionally regulated by auxin through several auxin response factors [67]. Apart from stimulation of root cell elongation, this mechanism is also involved in formation the lysigenous aerenchyma in rice roots in hypoxia conditions [68]. NADPH oxidases may not be the only system for ROS generation in root cell growth. Peroxidases can also participate in this process [69].

Outer layers of cells in different plants organs, such as guard cells, leaf and root epidermis, and particularly in the root tip, are the first to sense new environments. While the root elongates, the root tip explores new areas providing first contact with stresses as well. Internal tissues, such as the pericycle or vascular system, do not have contact with the soil and may not be involved in primary stress signalling. Supporting this, HO^•^-activated Ca^2+^ influx currents in root elongation zone and tips of root hairs was larger than in other tissues [12]. For example, it was 10 to 12 times larger in elongation zone cells than in cells of pericycle [12]. This indicates that Ca^2+^-permeable conductances responding to HO^•^ are necessary for both growth and sensing a new environment. Sidedness of ROS application, developmental stage of cells and potential source of generation are major factors altering activation of cation-permeable conductances in the root cell plasma membrane [5,18]. In contrast to the guard cells, H_2_O_2_ was unable to activate currents in mature *Arabidopsis thaliana* root epidermal cells when it was applied inside and outside the pipette in a whole-cell patch-clamp configuration or added outside in excised outside-out patches [12,18]. However, H_2_O_2_ activated Ca^2+^-permeable channels when it was applied to excised outside-out patches at the cytoplasmic side. This shows that H_2_O_2_ should be delivered directly to the channel inside mature epidermal cells. Nevertheless, in protoplasts from young cells, exogenously applied H_2_O_2_ was capable of activating Ca^2+^ currents [18]. This suggests that ROS-activated channels in different tissues/cells may be encoded by different genes or that younger root cells (and guard cells) may have a higher density of H_2_O_2_-permeable aquaporins which facilitate H_2_O_2_ delivery to the cytosol [70,71,72,73]. Hypothetically, elongating root cells and guard cells can have higher catalytic activities of transition metal ions converting H_2_O_2_ to hydroxyl radicals than mature root epidermal cells.

Apart from *Arabidopsis thaliana*, ROS-activated Ca^2+^-permeable channels were investigated in detail in *Pisum sativum* mature root cells [21]. In patch-clamped root protoplasts of this species, hydroxyl radicals but not H_2_O_2_ activated a Ca^2+^-permeable nonselective conductance, which was sensitive to cation channel blockers (Gd^3+^, nifedipine and verapamil) as well as to anion channel blockers (5-nitro-2(3-phenylpropylamino)-benzoate and niflumate). Interestingly, the same pharmacology was seen on K^+^ efflux and Ca^2+^ influx of HO^•^-treated intact pea roots (analysed by MIFE system). These authors also tested effect of polyamines on HO^•^-activated K^+^ and Ca^2+^ conductances. Polyamines are key endogenous stress protectants in higher plants. Their levels increase under stress conditions, such as salinity or drought and facilitate adaptation [74]. Intriguingly, polyamines (spermine, spermidine, and putrescine) stimulated the HO^•^-induced Ca^2+^ and K^+^ currents and fluxes in pea roots [21]. Authors of this study suggested that polyamines directly (chemically) interact with ROS increasing their reactivity. Hypothetically, polyamines can interact with ROS-activated channels by penetrating the pore and keeping it in open state that can be important for ROS-induced activation. Recent data showed that ROS-sensing groups in K^+^ channels activated by ROS become accessible to extracellular substances only when the channel is activated by depolarisation [75]. In the case of ROS-activated Ca^2+^-permeable channels reported by Zepeda-Jazo et al. [21], the accessibility of ROS-sensing group probably increases, when polyamines interact with the pore of the channel complex. Moreover, it should also be considered that polyamine radicals could have a longer half-life than HO^•^.

Single-channel characteristics of ROS-activated cation channels have been reported twice [13,18]. Hydrogen peroxide-activated Ca^2+^ influx unitary conductance was 14.5 pS (bath: 20 mM CaCl_2_; pipette: 25 mM Kgluconate, 5 mM KCl, 1 mM H_2_O_2_) [18]. The ROS-activated K^+^ channel unitary conductance was 16 pS (bath: 1 mM KCl, 0.3 mM CaCl_2_; pipette: 70 mM Kgluconate, 10 mM KCl) [13]. These data indicate that ROS activated one Ca^2+^-permeable channel and one K^+^-permeable channel. The gene of K^+^ channel activated by ROS was identified as *GORK*.

## 6. Structure and Function of ROS-Activated K^+^ Efflux Channels

Although major focus of ROS signaling in relation to ion channel activities has always been the Ca^2+^ influx, which is involved in growth, signalling and stress responses, another group of ROS-evoked events, which are related to ROS-activated K^+^ efflux channels, may be as important. The activation of K^+^ efflux channels by HO^•^ was demonstrated under salt stress conditions and pathogen attack [13]. Tests with electron paramagnetic resonance spectroscopy and the range of electrophysiological techniques showed that treatment of intact roots by high (NaCl) (100 and 250 mM) stimulated HO^•^ production leading to K^+^ channel activation and K^+^ efflux from roots [13]. Hydroxyl radicals and NaCl caused programmed cell death (PCD) and collapse of membrane potential in root cells of *Arabidopsis thaliana* in a K^+^-dependent manner (blocked by TEA^+^). These effects were delayed in plants, lacking functional K^+^ efflux channel AtGORK (*Atgork1-1*). *Atgork1-1* plants showed no K^+^ efflux (measured by MIFE), nor K^+^ outwardly-directed currents (measure by patch-clamp) in response to HO^•^. They also demonstrated much smaller K^+^ efflux after exposure to high NaCl levels, pathogen elicitors, hypoxia and other treatments [13,76]. GORK transcription was up-regulated upon onset of drought, salt stress and cold [77]. This effect was probably related to ROS production caused by stresses, because the level of GORK channel transcript significantly increased in the presence of O_2_^•−^ leading to an increased activity of this channel [78]. Identification of root HO^•^-activated K^+^ efflux channels (GORK) has linked stress induced ROS generation and electrolyte/K^+^ leakage [12,13]. Electrolyte leakage is a hallmark of stress in plant physiology [14]. However, until recently the mechanism of this phenomenon was not understood. According to the recently proposed metabolic adjustment hypothesis, K^+^ leakage mediated by GORK could also play the role of a ‘metabolic switch’, which decreases the rate of anabolic reactions and stimulates catabolic processes, causing the release of energy for adaptation and repair needs [14]. 

The HO^•^-activated K^+^ efflux mediated by GORK channels also explains how stress-induced ROS generation leading to K^+^/electrolyte leakage triggers PCD and autophagy. Demidchik et al. [13] demonstrated that K^+^ loss by root cells dramatically enhances the activities of endonucleases and proteases. This was accompanied by a set of cytological PCD symptoms. In animal cells, K^+^-dependent caspases and endonucleases are major players in PCD [79]. They are directly inhibited by high cytosolic K^+^ concentrations, but K^+^ loss through K^+^-permeable ion channels can release their activities [80]. Similar to plants, the cytoplasmic K^+^/Na^+^ ratio is a major parameter regulating animal PCD [81,82]. Sodium ions cannot substitute for K^+^ in its protease inhibition reaction therefore high Na^+^ levels ultimately lead to onset of PCD [80,81]. Various death factors can stimulate animal K^+^ efflux channels promoting increased protease and endonuclease activities [83]. Identification of ROS-activated K^+^ efflux channels in plants and characterisation of its role in PCD demonstrates that a similar mechanism of hydrolase activation exists in plants. K^+^-modulated PCD is also shared by fungi [84,85].

## 7. Pollen Tube ROS-Activated Channels

**Figure 1:**
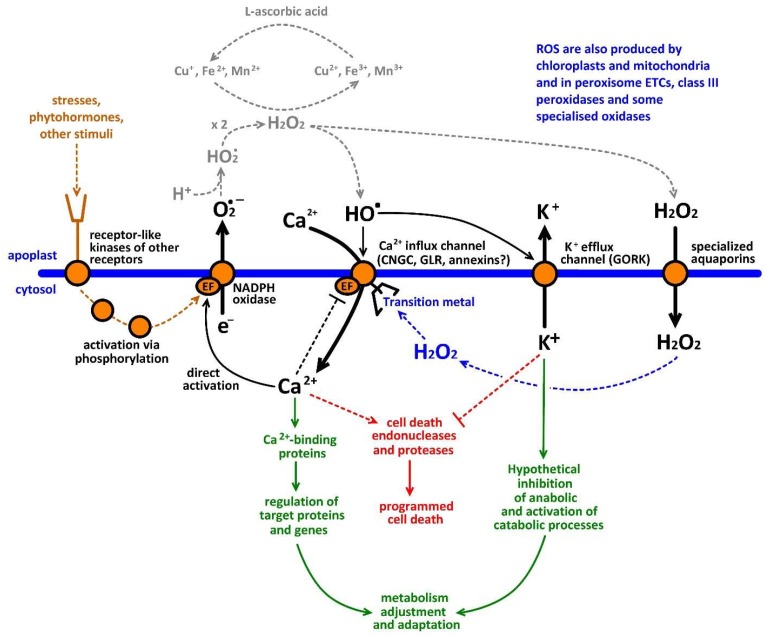
The concept of reactive oxygen species (ROS)-Ca^2+^-hub in the plasma membrane of higher plants. Stresses and regulatory stimuli can react with cell surface receptors leading to the activation of NADPH-oxidases (or directly activate ion channels). NADPH oxidases produce superoxide (O_2_^•−^), which binds H^+^ and forms hydroperoxyl radicals (HO^•^_2_), which undergo dismutation reaction forming H_2_O_2_. H_2_O_2_ can be reduced in Haber-Weiss cycle using electrons from transition metals, which can be reduced by l-ascorbic acid. H_2_O_2_ reduction leads for formation of HO^•^ in the close proximity (clusters) to ROS-activated Ca^2+^-permeable cation channels. Cell wall transition metals are required for Haber-Weiss cycle in the apoplast. Channels are activated in response to increased HO^•^ production and mediate Ca^2+^ influx and K^+^ efflux. Hydrogen peroxide can hypothetically activate channels from inside via reaction with transition metal binding sites (via generation of HO^•^). ROS-induced cytosolic Ca^2+^ elevation results in activation of signaling and regulatory cascades, stimulation of exocytosis and growth as well as induction of programmed cell death. ROS-activated efflux of K^+^ can lead to stimulation of programmed cell death, autophagy or metabolic adjustment, which is required for changing plant metabolism during stress responses and releasing energy for reparation needs [14].

Pollen tubes elongate using similar ion channel-mediated mechanism as root hairs (Figure 1). This mechanism is based on polar (apical) ROS-activated loading of Ca^2+^, which induces cytoskeleton rearrangement facilitating exocytosis and delivery of new cell wall material to the growing part of the cell [2,67,86]. Interestingly, growth-related gene expression is shared between pollen tubes and root hairs [77]. Similar to root hairs, a steep Ca^2+^ activity gradient in the tip of elongating pollen tubes is regulated via ROS produced by the NADPH oxidase [87]. Wu et al. [57] demonstrated that exogenous H_2_O_2_ rapidly activated plasma membrane inwardly-rectifying Ca^2+^-permeable conductance in pear pollen tube protoplasts. Breygina et al. [88] found very similar H_2_O_2_-activated conductances in lily pollen grain protoplasts. Breygina et al. [88] also demonstrated activation of weak K^+^ efflux current by H_2_O_2_ in lily pollen tube, also similar to root cells. Among the putative Ca^2+^-permeable ion channels, cyclic nucleotide-gated channels (CNGC7, CNGC8, CNGC16, and CNGC18), ionotropic glutamate receptors (GLR1.2, GLR1.4, GLR3.4, GLR3.7), ‘Reduced hyperosmolarity-induced [Ca^2+^]_i_ increase 1’ (OSCA1) and ‘Mechanosensitive channel of small conductance-like 8’ (MSL8) are expressed in pollen tube [86]. Moreover, a number of K^+^ channels, potentially including stellar K^+^ outward rectifier (SKOR), which can be activated by ROS, are expressed in the pollen tube [86]. Nevertheless, a direct link between gene and function is still missing in the case of ROS-activated ion channels in this important model. Recent studies showed that the mechanosensitive OSCA1 channel, which is responsible for Ca^2+^ entry to the cytosol due to osmolarity changes, is insensitive to H_2_O_2_ [89].

## 8. Annexins as Potential ROS-Activated Ion Channels

Annexins are proteins, which are normally located in the cytosol. However, in the presence of Ca^2+^, they are capable of association with membrane phospholipids and changing their characteristics of biomembranes. Eight putative genes encoding annexins have been identified in *Arabidopsis thaliana*, and 11 and 25 genes were found in barley and wheat, respectively [90]. Plant annexins consist of repeated annexin domains with a conserved endonexin fold binding Ca^2+^. Their structure significantly differs from animal annexins. Some authors proposed that annexins can form Ca^2+^ influx channels, which are regulated by ROS [91,92,93]. This is based on the observation that an addition of purified plant annexin protein induced elevation of cytosolic free Ca^2+^ in *Arabidopsis* root epidermal protoplasts, indicative of formation of ion channel-like Ca^2+^-permeable pores [91]. Laohavisit et al. [92] demonstrated that Ca^2+^ influx current activated by HO^•^ were lost in annexin KO mutants (*ann1*) lacking functional annexin. However, in should be noted that *ann1* plants are dwarfs with dramatically changed morphology and physiology. It is expected that characteristics of ion channels can be different in these plants compared to the wild type.

In animals, annexins are peripheral membrane proteins interacting with membranes in a Ca^2+^-dependent manner [94]. Ion channel activity of various animal annexins has been shown in artificial lipid bilayers while in vivo evidence for this is still missing. Animal annexin monomers cannot expand the lipid bilayer of the membrane. Animal annexins A7 and A5 trigger Ca^2+^ influx activity in the plasma membrane and endomembranes. One hypothetical mechanism suggests that annexins can assembly to a seven- or four-domain structure forming a transmembrane hydrophilic cation-permeable channel. At the same time, an alternative hypothesis proposed that a membrane destabilisation and electroporation as well as membrane flippage and resealing due to peripheral interaction with phospholipids underlie annexin function [94,95]. Annexins are usually associated with the onset of Ca^2+^-dependent apoptosis in different animal tissues. However recent studies showed that annexin action on apoptosis is induced by inhibition of K^+^ efflux channels [96]. In resting conditions, annexin blocked Ca^2+^-activated cytoplasmic site of K^+^ efflux channels, but increased cytosolic Ca^2+^ causes relocation of annexin to the extracellular side triggering K^+^ efflux channel activation by cytosolic Ca^2+^.

Wang et al. (2015) have recently found that Annexin1 was expressed in vesicles derived from the endoplasmic reticulum [97]. This expression was rapidly up-regulated by heat shock. KO plants lacking microsomal annexin showed abolished heat stress-induced [Ca^2+^]_cyt._ elevation and an increased heat sensitivity. Consistent with this, some animal annexins act as scaffolding proteins in microsomes. They anchor other proteins to the cell membrane and make connections between membranes. Plant annexins probably share this function and can hypothetically mediate intracellular trafficking of Ca^2+^-permeable channels [98]. Annexin knock-out lines may have less channel subunits delivered to the plasma membrane, demonstrating compromised Ca^2+^ signals [98]. Supporting this hypothesis, annexin knock-out mutants are dwarfed, having stunted growth and dramatically modified morphology [99]. Qiao et al. (2015) recently demonstrated that plant annexins, which have peroxidase domain, also generated H_2_O_2_ inside the cell and reacted with Ca^2+^-dependent protein kinases, promoting heat stress tolerance [100]. Thus, apart from the proposed direct insertion into the plasma membrane [91,92,101], intracellular annexins are likely affecting the delivery of Ca^2+^-permeable channels to the plasma membrane [98].

## 9. Mechanisms of Ion Channel Activation by ROS

The study by Garcia-Mata et al. [75] has shed light on the molecular mechanism of ROS-induced activation of K^+^ channels. Using heterologous expression systems (HEK293 cells and *Xenopus* oocytes), these authors demonstrated that the K^+^ channel SKOR, which has very similar structure to GORK, is activated by H_2_O_2_ via targeted oxidation of Cys168 at the S3 α-helix within channel’s voltage sensor. This residue is exposed to the outside when the GORK channel is in the open conformation. Substitution of this amino acid abolished SKOR’s sensitivity to H_2_O_2_. A corresponding Cys residue exists in GORK [75].

A mechanism of HO^•^ generation from H_2_O_2_ in the cation channel (leading to the channel activation) was proposed by Demidchik et al. [14]. Using Metal Detector ver. 2.0 software (Universities of Florence and Trento, Florence, Italy) the putative Cu/Fe binding sites in CNGC19 and CNGC20 were identified. Cys 102, 107 and 110 of CNGC19 and Cys 133, 138 and 141 of CNCG20 can co-ordinate transition metals assembling into the metal-binding sites with a probability close to 100%. These sites potentially generate HO^•^ from H_2_O_2_ within a channel complex, which is crucial, considering that HO^•^ is extremely short-lived and can therefore act at a distance not more than 1 nm from the point of its generation. Notably, cysteine residues have been shown to be responsible for direct HO^•^-induced activation of animal Ca^2+^-permeable channels [102] and transcription factors [103].

## 10. The Hypothesis of a ROS-Ca^2+^ Hub for Amplification of Redox and Ca^2+^ Signals at the Plant-Environment Interface

ROS are critically important for stress and hormonal signalling, polar and gravitropic growth, autophagy, PCD and other functions of higher plants [29,104]. ROS and cytosolic Ca^2+^ signals simultaneously control the same physiological processes and cross-talk via reciprocal stimulation—Ca^2+^-dependent activation of NADPH oxidase and ROS activation of Ca^2+^-permeable channels (Table 1).

The hypothesis of a ‘ROS-Ca^2+^ hub’ (Figure 1) assumes that the Ca^2+^-activated NADPH oxidases (encoded by RBOH genes) act together with ROS-activated Ca^2+^-permeable channels to amplify stress-induced Ca^2+^ and ROS signals [2,12,18,29,104]. Elevation of cytosolic Ca^2+^ level causes an increase in superoxide production and vice versa, superoxide, via generation HO^•^, activates ROS-activated Ca^2+^ influx channels (Figure 1). This forms a self-amplification positive feedback loop stimulating duration and amplitude of initially weak signals and transforming them into the large-scale responses [58]. Moreover, this also involves higher activation of GORK-mediated K^+^ efflux [13]. The analysis of recent literature demonstrated that ROS-Ca^2+^ hub is involved in root and pollen tube growth, hypersensitive response to pathogen, transduction of hormonal and other regulatory chemical signals, responses to abiotic stresses, water balance, autophagy, programmed cell death and mineral nutrition [5]. The ROS-Ca^2+^ has a sophisticated system for control and regulation in plants, which include self-inactivation of Ca^2+^-permeable channels, induction of Ca^2+^-ATPase through Ca^2+^-calmodulin binding to specific binding sites of these enzymes, suppression of Rac/Rop GTPases, and Ca^2+^-Dependent Protein Kinase (CDPK)-catalysed phosphorylation of ion channels or Botrytis-Induced Kinase (BIK)1-catalysed phosphorylation of NADPH oxidase [5,104,105,106,107,108,109,110,111,112]. 

The concept of ROS-Ca^2+^ hub allows to predict the ion channel subunits involved in activation by ROS [5]. Most ion channels activated by ROS have not yet been identified genetically or electrophysiologically. However, there is indirect evidence that they function in concert with some specific NADPH oxidases (Table 1). An involvement of both NADPH oxidase and channels points to potential activation of channels by ROS. One of the widely acknowledged examples of ‘ROS-Ca^2+^ hub’ function is the loading of Ca^2+^ for elongation growth of root cells [6,12] and pollen tubes [87]. This process is driven by extremely high polar NADPH oxidase activity leading to activation of Ca^2+^-permeable channels. ROS-Ca^2+^ hub maintains [Ca^2+^]_cyt_ in growing part of the cell, such as tips root hair of pollen tube, at constantly high level, supporting longitudinal cytoskeleton bundles and tip-directed exocytosis [105]. *Arabidopsis* CNGC3 [113], annexins [92] and RbohC [6] probably form the root hair ROS-Ca^2+^ hub. Ionotropic glutamate receptors GLR1.2 and GLR3.7 [86] and CNGC18 [114], jointly with RbohH and RbohJ [115] could function as a ROS-Ca^2+^ hub in *Arabidopsis* pollen tube. 

The ROS-Ca^2+^ hub is also involved in plant cell hypersensitive response during pathogen attack, leading to a massive PCD around the infection site and probably preventing spread of the disease [116]. AtrbohD and AtrbohF [117,118] may cross-talk with AtCNGC2, 4, 11 and 12 [119] to form ‘ROS-Ca^2+^ hub’ mediating this reaction. The following hormones are potentially involved in the ROS-Ca^2+^ hub: abscisic acid (AtrbohD and AtrbohF cross-talking with AtCNGC5 and AtCNGC6) [53], auxins (AtRbohD/AtCNGC14) [120,121,122], methyl jasmonate (AtrbohD and AtrbohF/AtCNGC2) [123,124] and salicylic acid (AtrbohD/AtGLR3.3) [125,126,127]. Drought induces stomata closure via abscisic acid-mediated activation of AtrbohD and AtrbohF/AtCNGC5 and AtCNGC6 couples [128]. Gémes et al. (2016) have shown that NtRbohD and NtRbohF are crucial for NaCl-induced ROS production and activation of ROS-Ca^2+^ hub [129], while Guo et al. (2008, 2010) demonstrated that Ca^2+^ entry in this case is mediated by AtCNGC10 [130,131]. High temperatures are directly sensed via membrane fluidity changes by CNGC6 [113]. At the same time the heat tolerance is impaired in AtrbohD and AtrbohB KO lines, suggesting a clash with CNGC6 [132]. Overall, these data strongly suggest that the ‘ROS-Ca^2+^ hub’ is an important and ubiquitous mechanism, which is potentially involved in a multitude of physiological processes.

## 11. Summary and Concluding Remarks

Key biophysical and functional properties of ROS-activated ion channels are summarized in Table 2. Conductances mediated by these channels have been measured in the plasma membrane of Charophyte *Nitella flexilis*, three dicotyledous higher plants, including *Arabidopsis thaliana*, *Pyrus pyrifolia* and *Pisum sativum*, and one monocotyledon *Lilium longiflorum*. They exist in root hairs, root mature and elongation zone epidermal cells, pollen tubes, and guard cells. Activating species include H_2_O_2_ and hydroxyl radicals. Prevalent intracellular activating species is H_2_O_2_ while, in the extracellular medium, it is the hydroxyl radicals. ROS-activated ion channels can be divided into the following three groups by the shape of current-voltage curves: inwardly-rectifying, voltage-independent and outwardly-rectifying. Channels of inwardly-rectifying group conducts Ca^2+^-influx currents for growth and signaling needs, while the outwardly-rectifying members transport K^+^ from the cell to extracellular medium to regulate osmotic pressure for guard cell closure, induce programmed cell death or autophagy, or hypothetically adjust metabolism for reparation needs [14]. The voltage-independent group is less studied, but it mediates both Ca^2+^-influx and K^+^ efflux, co-existing with other types of conductances in same patches. Selectivity studies are contradicting, showing that ROS-activated channels could be fully nonselective, including permeation to anions, or can specifically conduct only cations. Most reports suggest that ROS-activated channels are cation-selective. However further studies on selectivity are definitely needed. *Arabidopsis* Ca^2+^-permeable ROS-activated channels are blocked by lanthanides, verapamil and nifedipine, while the same channels in pea are also sensitive to some anion channel blockers, 5-nitro-2(3-phenylpropylamino)-benzoate and niflumate. ROS-activated K^+^-permeable channels are blocked by TEA^+^. Time-dependence of activation can also vary including rapidly- and slowly-activated channels. Both Ca^2+^ influx and K^+^ efflux ROS-activated conductances include rapidly- and slowly-activated current components. Few preparations showed single-channel characteristics revealing 14.5-pS Ca^2+^ influx and 16-pS K^+^ efflux ROS-activated unitary conductances. Molecular nature of ROS-activated conductances is defined only for K^+^ efflux channels, which are encoded by SKOR and GORK genes, both having ROS sensing cysteine moieties. Moreover, more genes can be predicted from the analysis of NADPH oxidase-mediated ROS-Ca^2+^-hubs. Established physiological functions of ROS-activated conductances include root cell and pollen tube growth, regulation of osmotic balance and stomata aperture, responses to major stresses, amplification of external stress and phytohormonal signals via ROS-Ca^2+^-hub, programmed cell death, and autophagy. Undoubtedly, this list will be extended in further studies on ROS-activated ion channels.

## Figures and Tables

**Table 1:** NADPH oxidases and cation channels, which can potentially function as ROS-Ca^2+^ hubs in key physiological reactions (see hypothesis on ROS-Ca^2+^ hubs in the text).

Physiological Process	Genes of Cation Channels with Predicted or Demonstrated Ca^2+^ Permeability	Genes Encoding NADPH Oxidase Producing ROS for Activation of Ca^2+^-Permeable Channels	References
Growth and development			
Root cell elongation	AtCNGC3	AtRBOHC	[6,113]
Pollen tube elongation	AtCNGC18, AtGLR1.2, AtGLR3.7	AtRBOHH, AtRBOHJ	[86,115,133]
Phytohormonal regulation			
Effects of auxin	AtCNGC14	AtRBOHD	[120,121,122]
Abscisic acid signalling	AtCNGC5, AtCNGC6	AtRBOHD, AtRBOHF	[7,122]
Methyl jasmonate-induced reactions	AtCNGC2	AtRBOHD, AtRBOHF	[123,124]
Action of salicylic acid	AtGLR3.3	AtRBOHD	[125,126,127]
Stress responses			
Hypersensitive response (massive PCD around the spot of infection, preventing spread of the disease)	AtCNGC2, AtCNGC4, AtCNGC11, AtCNGC12	AtRBOHD, AtRBOHF	[117,118,119]
Response to salinity	AtCNGC10	NtRBOHD, NtRBOHF	[128,130]
Drought-induced stomata closure	AtCNGC5, AtCNGC6	AtRBOHD, AtRBOHF	[128]
Response to extreme temperatures	AtCNGC6	AtRBOHD, AtRBOHB	[114,132]

**Table 2:** Biophysical and functional characteristics of ROS-activated ion channels in plant plasma membranes.

Preparation	Activating ROS	Selectivity and Kinetics of Activation	Blockers, Modulators	Function	References
*Nitella flexilis*					
- intermodal cells	Cu^2+^, HO^•^	NS *, VI *, RA *	Lanthanides, verapamil, nifedipine	Sensing transition metals, copper toxicity	[17,22]
*Arabidopsis thaliana*					
- root hairs	HO^•^	NS, IR *, SA *	Lanthanides	Growth	[6,12]
- root mature epidermis	HO^•^, H_2_O_2_	NS, IR, SA	Lanthanides, verapamil TEA^+^	Stress response	[12,18]
- root elongation zone	HO^•^	KS *, OR *, SA NS, VI, RA	Lanthanides, verapamil	Growth, stress response	[12]
- guard cells	H_2_O_2_ H_2_O_2_	KS, OR, SA NS, VI, RA NS, IR	Lanthanides Lanthanides, verapamil	ABA signaling, stomata closure	[10]
*Pyrus pyrifolia*					
- pollen tube	H_2_O_2_	IR	Activation by polyamines	Pollen tube growth	[57]
*Pisum sativum*					
- root	HO^•^	NS, VI, RA	Stimulation by polyamines		[21]
*Lilium longiflorum*					
- pollen tube	H_2_O_2_	NS, IR, SA KS, OR, SA	Lanthanides, nifedipine TEA^+^	Pollen tube growth	[88]

* NS—non-selective, RA—rapidly-activating, SA—slowly-activating, VI—voltage-independent, OR—outwardly-rectifying, IR—inwardly-rectifying, KS—K^+^-selective.

## References

[1] Tester M. (1990). Plant ion channels: Whole-cell and single channel studies. New Phytol..

[2] Demidchik V., Maathuis F.J.M. (2007). Physiological roles of nonselective cation channels in plants: From salt stress to signalling and development. New Phytol..

[3] Kollist H., Jossier M., Laanemets K., Thomine S. (2011). Anion channels in plant cells. FEBS J..

[4] Hedrich R. (2012). Ion channels in plants. Physiol. Rev..

[5] Demidchik V., Shabala S. (2017). Mechanisms of cytosolic calcium elevation in plants: The role of ion channels, calcium extrusion systems and NADPH oxidase-mediated ‘ROS-Ca^2+^ Hub’. Funct. Plant Biol..

[6] Foreman J., Demidchik V., Bothwell J.H.F., Mylona P., Miedema H., Torres M.A., Linstead P., Costa S., Brownlee C., Jones J.D. (2003). Reactive oxygen species produced by NADPH oxidase regulate plant cell growth. Nature.

[7] Wang Y.F., Fan L.M., Zhang W.Z., Zhang W., Wu W.H. (2004). Ca^2+^-permeable channels in the plasma membrane of *Arabidopsis* pollen are regulated by actin microfilaments. Plant Physiol..

[8] Jurkowski G.I., Smith R.K., Yu I.C., Ham J.H., Sharma S.B., Klessig D.F., Fengler K.A., Bent A.F. (2004). *Arabidopsis* DND2, a second cyclic nucleotide-gated ion channel gene for which mutation causes the “defense, no death” phenotype. Mol. Plant Microbe Interact..

[9] Ache P., Becker D., Ivashikina N., Dietrich P., Roelfsema M.R., Hedrich R. (2000). GORK, a delayed outward rectifier expressed in guard cells of *Arabidopsis thaliana*, is a K^+^-selective, K^+^-sensing ion channel. FEBS Lett..

[10] Pei Z.M., Murata Y., Benning G., Thomine S., Klusener B., Allen G.J., Grill E., Schroeder J.I. (2000). Calcium channels activated by hydrogen peroxide mediate abscisic acid signalling in guard cells. Nature.

[11] Hosy E., Vavasseur A., Mouline K., Dreyer I., Gaymard F., Poree F., Boucherez J., Lebaudy A., Bouchez D., Very A.A. (2003). The *Arabidopsis* outward K^+^ channel GORK is involved in regulation of stomatal movements and plant transpiration. Proc. Natl. Acad. Sci. USA.

[12] Demidchik V., Shabala S.N., Coutts K.B., Tester M.A., Davies J.M. (2003). Free oxygen radicals regulate plasma membrane Ca^2+^- and K^+^-permeable channels in plant root cells. J. Cell Sci..

[13] Demidchik V., Cuin T.A., Svistunenko D., Smith S.J., Miller A.J., Shabala S., Sokolik A., Yurin V. (2010). *Arabidopsis* root K^+^ efflux conductance activated by hydroxyl radicals: Single-channel properties, genetic basis and involvement in stress-induced cell death. J. Cell Sci..

[14] Demidchik V., Straltsova D., Medvedev S.S., Pozhvanov G.A., Sokolik A., Yurin V. (2014). Stress-induced electrolyte leakage: The role of K^+^-permeable channels and involvement in programmed cell death and metabolic adjustment. J. Exp. Bot..

[15] Herdean A., Teardo E., Nilsson A.K., Pfeil B.E., Johansson O.N., Ünnep R., Nagy G., Zsiros O., Dana S., Solymosi K. (2016). A voltage-dependent chloride channel fine-tunes photosynthesis in plants. Nat. Commun..

[16] Checchetto V., Teardo E., Carraretto L., Formentin E., Bergantino E., Giacometti G.M., Szabo I. (2013). Regulation of photosynthesis by ion channels in cyanobacteria and higher plants. Biophys. Chem..

[17] Demidchik V., Sokolik A., Yurin V. (1997). The effect of Cu^2+^ on ion transport systems of the plant cell plasmalemma. Plant Physiol..

[18] Demidchik V., Shabala S., Davies J. (2007). Spatial variation in H_2_O_2_ response of *Arabidopsis thaliana* root epidermal Ca^2+^ flux and plasma membrane Ca^2+^ channels. Plant J..

[19] Demidchik V., Shang Z., Shin R., Shabala S., Davies J.M. (2011). Receptor-like activity evoked by extracellular ADP in *Arabidopsis thaliana* root epidermal plasma membrane. Plant Physiol..

[20] Yuan F., Yang H., Xue Y., Kong D., Ye R., Li C., Zhang J., Theprungsirikul L., Shrift T., Krichilsky B. (2014). OSCA1 mediates osmotic-stress-evoked Ca^2+^ increases vital for osmosensing in *Arabidopsis*. Nature.

[21] Zepeda-Jazo I., Velarde-Buendía A.M., Enríquez-Figueroa R., Bose J., Shabala S., Muñiz-Murguía J., Pottosin I.I. (2011). Polyamines interact with hydroxyl radicals in activating Ca^2+^ and K^+^ transport across the root epidermal plasma membranes. Plant Physiol..

[22] Demidchik V., Sokolik A., Yurin V. (2001). Characteristics of non-specific permeability and H^+^-ATPase inhibition induced in the plasma membrane of Nitella flexilis by excessive Cu^2+^. Planta.

[23] Demidchik V., Maathuis F.J.M. (2010). Ion Channels and Plant Stress Responses.

[24] Murata Y., Mori I.C., Munemasa S. (2015). Diverse stomatal signaling and the signal integration mechanism. Annu. Rev. Plant Biol..

[25] Halliwell B., Gutteridge J.M.C. (2015). Free Radicals in Biology and Medicine.

[26] Fenton H.J.H. (1894). Oxidation of tartaric acid in presence of iron. J. Chem. Soc. Trans..

[27] Haber F., Weiss J. (1932). On the catalysis of hydroperoxide. Naturwissenschaften.

[28] Goldstein S., Meyerstein D., Czapski G. (1993). The Fenton reagents. Free Radic. Biol. Med..

[29] Demidchik V. (2015). Mechanisms of oxidative stress in plants: From classical chemistry to cell biology. Environ. Exp. Bot..

[30] McBrien D.C.H., Hassall K.A. (1965). Loss of cell potassium by *Chlorella vulgaris* after contact with toxic amounts of copper sulphate. Physiol. Plant.

[31] Overnell J. (1975). The effect of heavy metals on photosynthesis and loss of cell potassium in two species of marine algae, *Dunaliella tertiolecta* and *Phaeodactylum tricomutum*. Mar. Biol..

[32] De Filippis L.F. (1979). The effect of heavy metal compounds on the permeability of Chlorella cells. Z. Pflanzenphysiol..

[33] Wainwright S.J., Woolhouse H.W. (1977). Some physiological aspects of copper and zinc tolerance in *Agrostis tenuis* Sibth: Cell elongation and membrane damage. J. Exp. Bot..

[34] De Vos C., Schat H., Vooijs R., Ernst W. (1989). Copper induced damage to the permeability barrier in roots of *Silene cucubalus*. J. Plant Physiol..

[35] Luna C., Gonzalez C., Trippi V. (1994). Oxidative damage caused by an excess of copper in oat leaves. Plant Cell Physiol..

[36] Murphy A., Taiz L. (1997). Correlation between potassium efflux and copper sensitivity in ten *Arabidopsis* ecotypes. New Phytol..

[37] Murphy A.S., Eisinger W.R., Shaff J.E., Kochian L.V., Taiz L. (1999). Early copper-induced leakage of K^+^ from *Arabidopsis* seedlings is mediated by ion channels and coupled to citrate efflux. Plant Physiol..

[38] Palta J.P., Levitt J., Stadelmann E.J. (1977). Freezing injury in onion bulb cells. I. Evaluation of the conductivity method and analysis of ion and sugar efflux from injured cells. Plant Physiol..

[39] Shabala S. (2017). Plant Stress Physiology.

[40] Atkinson M.M., Midland S.L., Sims J.J., Keen N.T. (1996). Syringolide 1 triggers Ca^2+^ influx, K^+^ efflux, and extracellular alkalization in soybean cells carrying the disease-resistance gene Rpg4. Plant Physiol..

[41] Atkinson M.M., Huang J.S., Knopp J.A. (1985). The hypersensitive response of tobacco to *Pseudomonas syringae* pv. pisi: Activation of a plasmalemma K^+^/H^+^ exchange mechanism. Plant Physiol..

[42] Atkinson M.M., Keppler L.D., Orlandi E.W., Baker C.J., Mischke C.F. (1990). Involvement of plasma membrane calcium influx in bacterial induction of the K^+^/H^+^ and hypersensitive responses in tobacco. Plant Physiol..

[43] Van Dongen J.T., Licausi F. (2015). Oxygen sensing and signaling. Annu. Rev. Plant Biol..

[44] Levitt J. (1972). Responses of Plants to Environmental Stresses: Chilling, Freezing and High Temperature Stresses v. 1 (Physiological ecology).

[45] Nassery H. (1975). The effect of salt and osmotic stress on the retention of Potassium by excised barley and bean roots. New Phytol..

[46] Nassery H. (1979). Salt-induced loss of potassium from plant roots. New Phytol..

[47] Blum A., Ebercon A. (1979). Cell membrane stability as a measure of drought and heat tolerance in wheat. Crop Sci..

[48] Leopold A.C., Musgrave M.E., Williams K.M. (1981). Solute leakage resulting from leaf desiccation. Plant Physiol..

[49] Price A.H. (1990). A possible role for calcium in oxidative plant stress. Free Radic. Res. Commun..

[50] Price A.H., Taylor A., Ripley S.J., Griffiths A., Trewavas A.J., Knight M.R. (1994). Oxidative signals in tobacco increase cytosolic calcium. Plant Cell.

[51] Clayton H., Knight M.R., Knight H., McAinsh M.R., Hetherington A.M. (1999). Dissection of the ozone-induced calcium signature. Plant J..

[52] Demidchik V.V., Sokolik A.I., Yurin V.M. (1996). Mechanisms of conductance modification in plant cell membranes under the action of trivalent iron ions. Doklady Akademii Nauk Belarusi.

[53] Mori I.C., Schroeder J.I. (2004). Reactive oxygen species activation of plant Ca^2+^ channels. a signaling mechanism in polar growth, hormone transduction, stress signaling, and hypothetically mechanotransduction. Plant Physiol..

[54] Meinhard M., Rodriguez P.L., Grill E. (2002). The sensitivity of ABI2 to hydrogen peroxide links the abscisic acid-response regulator to redox signaling. Planta.

[55] Meinhard M., Grill E. (2001). Hydrogen peroxide is a regulator of ABI1, a protein phosphatase 2C from *Arabidopsis*. FEBS Lett..

[56] Sierla M., Waszczak C., Vahisalu T., Kangasjärvi J. (2016). Reactive oxygen species in the regulation of stomatal movements. Plant Physiol..

[57] Wu H., Shabala L., Zhou M., Shabala S. (2015). Chloroplast-generated ROS dominate NaCl^−^ induced K^+^ efflux in wheat leaf mesophyll. Plant Signal. Behav..

[58] Demidchik V., Shang Z., Shin R., Thompson E., Rubio L., Laohavisit A., Mortimer J.C., Chivasa S., Slabas A.R., Glover B.J. (2009). Plant extracellular ATP signaling by plasma membrane NADPH oxidase and Ca^2+^ channels. Plant J..

[59] Fry S.C., Miller J.G., Dumville J.C. (2002). A proposed role for copper ions in cell wall loosening. Plant Soil.

[60] Bogeski I., Niemeyer B.A. (2014). Redox regulation of ion channels. Antioxid Redox Signal.

[61] Lebaudy A., Véry A.A., Sentenac H. (2007). K^+^ channel activity in plants: Genes, regulations and functions. FEBS Lett..

[62] Monshausen G.B., Bibikova T.N., Messerli M.A., Shi C., Gilroy S. (2007). Oscillations in extracellular pH and reactive oxygen species modulate tip growth of *Arabidopsis* root hairs. Proc. Natl. Acad. Sci. USA.

[63] Coelho S.M.B., Brownlee C., Bothwell J.H.F. (2008). A tip-high, Ca^2+^-interdependent, reactive oxygen species gradient is associated with polarized growth in *Fucus serratus* zygotes. Planta.

[64] Mendrinna A., Persson S. (2015). Root hair growth: It’s a one way street. F1000Prime Rep..

[65] Libik-Konieczny M., Kozieradzka-Kiszkurno M., Desel C., Michalec-Warzecha Ż., Miszalski Z., Konieczny R. (2015). The localization of NADPH oxidase and reactive oxygen species in in vitro-cultured *Mesembryanthemum crystallinum* L. hypocotyls discloses their differing roles in rhizogenesis. Protoplasma.

[66] Causin H.F., Roqueiro G., Petrillo E., Láinez V., Pena L.B., Marchetti C.F., Gallego S.M., Maldonado S.I. (2012). The control of root growth by reactive oxygen species in *Salix nigra* Marsh. seedlings. Plant Sci..

[67] Mangano S., Denita-Juarez S.P., Choi H.S., Marzol E., Hwang Y., Ranocha P., Velasquez S.M., Borassi C., Barberini M.L., Aptekmann A.A. (2017). Molecular link between auxin and ROS-mediated polar growth. Proc. Natl. Acad. Sci. USA.

[68] Yamauchi T., Yoshioka M., Fukazawa A., Mori H., Nishizawa N.K., Tsutsumi N., Yoshioka H., Nakazono M. (2017). An NADPH Oxidase RBOH functions in rice roots during Lysigenous aerenchyma formation under oxygen-deficient conditions. Plant Cell.

[69] Dunand C., Crèvecoeur M., Penel C. (2007). Distribution of superoxide and hydrogen peroxide in *Arabidopsis* root and their influence on root development: Possible interaction with peroxidases. New Phytol..

[70] Eisenbarth D.A., Weig A.R. (2005). Dynamics of aquaporins and water relations during hypocotyl elongation in *Ricinus communis* L. seedlings. J. Exp. Bot..

[71] Bienert G.P., Møller A.L., Kristiansen K.A., Schulz A., Møller I.M., Schjoerring J.K., Jahn T.P. (2007). Specific aquaporins facilitate the diffusion of hydrogen peroxide across membranes. J. Biol. Chem..

[72] Bienert G.P., Schjoerring J.K., Jahn T.P. (2006). Membrane transport of hydrogen peroxide. Biochim. Biophys. Acta.

[73] Vieceli Dalla Sega F., Zambonin L., Fiorentini D., Rizzo B., Caliceti C., Landi L., Hrelia S., Prata C. (2014). Specific aquaporins facilitate Nox-produced hydrogen peroxide transport through plasma membrane in leukaemia cells. Biochim. Biophys. Acta.

[74] Pottosin I., Shabala S. (2014). Polyamines control of cation transport across plant membranes: Implications for ion homeostasis and abiotic stress signaling. Front Plant Sci..

[75] Garcia-Mata C., Wang J., Gajdanowicz P., Gonzalez W., Hills A., Donald N., Riedelsberger J., Amtmann A., Dreyer I., Blatt M.R. (2010). A minimal cysteine motif required to activate the SKOR K^+^ channel of *Arabidopsis* by the reactive oxygen species H_2_O_2_. J. Biol. Chem..

[76] Wang F., Chen Z.H., Liu X., Colmer T.D., Shabala L., Salih A., Zhou M., Shabala S. (2016). Revealing the roles of GORK channels and NADPH oxidase in acclimation to hypoxia in *Arabidopsis*. J. Exp. Bot..

[77] Becker D., Hoth S., Ache P., Wenkel S., Roelfsema M.R., Meyerhoff O., Hartung W., Hedrich R. (2003). Regulation of the ABA-sensitive *Arabidopsis* potassium channel gene GORK in response to water stress. FEBS Lett..

[78] Tran D., El-Maarouf-Bouteau H., Rossi M., Biligui B., Briand J., Kawano T., Mancuso S., Bouteau F. (2013). Post-transcriptional regulation of GORK channels by superoxide anion contributes to increases in outward-rectifying K^+^ currents. New Phytol..

[79] Remillard C.V., Yuan J.X.-J. (2004). Activation of K^+^ channels: An essential pathway in programmed cell death. Am. J. Physiol. Lung Cell Mol. Physiol..

[80] Kondratskyi A., Kondratska K., Skryma R., Prevarskaya N. (2015). Ion channels in the regulation of apoptosis. Biochim. Biophys. Acta.

[81] Orlov S., Hamet P. (2006). JIntracellular monovalent ions as second messengers. Membr. Biol..

[82] Bortner C.D., Gómez-Angelats M., Cidlowski J.A. (2001). Plasma membrane depolarization without repolarization is an early molecular event in anti-fas-induced apoptosis. J. Biol. Chem..

[83] Yu S.P. (2003). Regulation and critical role of potassium homeostasis in apoptosis. Prog. Neurobiol..

[84] Lauff D.B., Santa-María G.E. (2010). Potassium deprivation is sufficient to induce a cell death program in *Saccharomyces cerevisiae*. FEMS Yeast Res..

[85] Yun J., Lee D.G. (2017). Role of potassium channels in chlorogenic acid-induced apoptotic volume decrease and cell cycle arrest in *Candida albicans*. Biochim. Biophys. Acta.

[86] Michard E., Simon A.A., Tavares B., Wudick M.M., Feijó J.A. (2017). Signaling with ions: The keystone for apical cell growth and morphogenesis in pollen tubes. Plant Physiol..

[87] Potocky M., Jones M.A., Bezvoda R., Smirnoff N., Zarsky V. (2007). Reactive oxygen species produced by NADPH oxidase are involved in pollen tube growth. New Phytol..

[88] Breygina M.A., Abramochkin D.V., Maksimov N.M., Yermakov I.P. (2016). Hydrogen peroxide affects ion channels in lily pollen grain protoplasts. Plant Biol..

[89] Makavitskaya M., Svistunenko D., Navaselsky I., Hryvusevich P., Mackievic V., Rabadanova C., Tyutereva E., Samokhina V., Straltsova D., Sokolik A. (2018). Novel roles of ascorbate in plants: induction of cytosolic Ca^2+^ signals and efflux from cells via anion channels. J. Exp. Bot..

[90] Xu L., Tang Y., Gao S., Su S., Hong L., Wang W., Fang Z., Li X., Ma J., Quan W. (2016). Comprehensive analyses of the annexin gene family in wheat. BMC Genom..

[91] Laohavisit A., Mortimer J.C., Demidchik V., Coxon K.M., Stancombe M.A., Macpherson N., Brownlee C., Hofmann A., Webb A.A., Miedema H. (2009). Zea mays annexins modulate cytosolic free Ca^2+^ and generate a Ca^2+^-permeable conductance. Plant Cell.

[92] Laohavisit A., Shang Z., Rubio L., Cuin T.A., Véry A.A., Wang A., Mortimer J.C., Macpherson N., Coxon K.M., Battey N.H. (2012). *Arabidopsis* annexin1 mediates the radical-activated plasma membrane Ca^2+^- and K^+^-permeable conductance in root cells. Plant Cell.

[93] Baucher M., Pérez-Morga D., El Jaziri M. (2012). Insight into plant annexin function: From shoot to root signaling. Plant Signal Behav..

[94] Lizarbe M.A., Barrasa J.I., Olmo N., Gavilanes F., Turnay J. (2013). Annexin-phospholipid interactions. Functional implication. Int. J. Mol. Sci..

[95] Carmeille R., Degrelle S.A., Plawinski L., Bouvet F., Gounou C., Evain-Brion D., Brisson A.R., Bouter A. (2015). Annexin-A5 promotes membrane resealing in human trophoblasts. Biochim. Biophys. Acta.

[96] Brazier S.P., Telezhkin V., Kemp P.J. (2016). Functional Interactions between BKCaα-subunit and annexin A5: Implications in apoptosis. Oxid. Med. Cell. Longev..

[97] Wang X., Ma X., Wang H., Li B., Clark G., Guo Y., Roux S., Sun D., Tang W. (2015). Proteomic study of microsomal proteins reveals a key role for *Arabidopsis* annexin 1 in mediating heat stress-induced increase in intracellular calcium levels. Mol. Cell. Proteom..

[98] Konopka-Postupolska D., Clark G. (2017). Annexins as overlooked regulators of membrane trafficking in plant cells. Int. J. Mol. Sci..

[99] Konopka-Postupolska D., Clark G., Goch G., Debski J., Floras K., Cantero A., Fijolek B., Roux S., Hennig J. (2009). The role of annexin 1 in drought stress in *Arabidopsis*. Plant Physiol..

[100] Qiao B., Zhang Q., Liu D., Wang H., Yin J., Wang R., He M., Cui M., Shang Z., Wang D., Zhu Z. (2015). A calcium-binding protein, rice annexin OsANN1, enhances heat stress tolerance by modulating the production of H_2_O_2_. J. Exp. Bot..

[101] Laohavisit A., Richards S.L., Shabala L., Chen C., Colaço R.D., Swarbreck S.M., Shaw E., Dark A., Shabala S., Shang Z. (2013). Salinity-induced calcium signaling and root adaptation in *Arabidopsis* require the calcium regulatory protein annexin. Plant Physiol..

[102] Simon L., Gauvin F., Amre D.K., Saint-Louis P., Lacroix J. (2004). Serum procalcitonin and C-reactive protein levels as markers of bacterial infection: A systematic review and meta-analysis. Clin. Infect Dis..

[103] Dubbs J.M., Mongkolsuk S. (2012). Peroxide-sensing transcriptional regulators in bacteria. J. Bacteriol..

[104] Shabala S., Wu H.H., Bose J. (2015). Salt stress sensing and early signalling events in plant roots: Current knowledge and hypothesis. Plant Sci..

[105] Noctor G., Reichheld J.P., Foyer C.H. (2017). ROS-related redox regulation and signaling in plants. Semin. Cell Dev. Biol..

[106] Baxter-Burrell A., Yang Z., Springer P.S., Bailey-Serres J. (2002). RopGAP4-dependent Rop GTPase rheostat control of *Arabidopsis* oxygen deprivation tolerance. Science.

[107] Tidow H., Poulsen L.R., Andreeva A., Knudsen M., Hein K.L., Wiuf C., Palmgren M.G., Nissen P. (2012). A bimodular mechanism of calcium control in eukaryotes. Nature.

[108] Zhou J., Wang J., Li X., Xia X.J., Zhou Y.H., Shi K. (2014). H_2_O_2_ mediates the crosstalk of brassinosteroid and abscisic acid in tomato responses to heat and oxidative stresses. J. Exp. Bot..

[109] Ben-Johny M., Yue D.N., Yue D.T. (2016). Detecting stoichiometry of macromolecular complexes in live cells using FRET. Nat. Commun..

[110] Kadota Y., Shirasu K., Zipfel C. (2015). Regulation of the NADPH Oxidase RBOHD during Plant Immunity. Plant Cell Physiol..

[111] DeFalco T.A., Moeder W., Yoshioka K. (2016). Opening the Gates: Insights into Cyclic Nucleotide-Gated Channel-Mediated Signaling. Trends Plant Sci..

[112] Rounds C.M., Bezanilla M. (2013). Growth mechanisms in tip-growing plant cells. Annu. Rev. Plant Biol..

[113] Gobert A., Park G., Amtmann А., Sanders D., Maathuis F.J.M. (2006). *Arabidopsis thaliana* cyclic nu leotide gated channel 3 forms a non-selective ion transporter involved in germination and cation transport. J. Exp. Bot..

[114] Gao Q.F., Gu L.L., Wang H.Q., Fei C.F., Fang X., Hussain J., Sun S.J., Dong J.Y., Liu H., Wang Y.F. (2016). Cyclic nucleotide-gated channel 18 is an essential Ca^2+^ channel in pollen tube tips for pollen tube guidance to ovules in *Arabidopsis*. Proc. Natl. Acad. Sci. USA.

[115] Kaya A., Lobanov A.V., Gerashchenko M.V., Koren A., Fomenko D.E., Koc A., Gladyshev V.N. (2014). Thiol peroxidase deficiency leads to increased mutational load and decreased fitness in *Saccharomyces cerevisiae*. Genetics.

[116] Levine A., Pennell R.I., Alvarez M.E., Palmer R., Lamb C. (1996). Calcium mediated apoptosis in a plant hypersensitive disease resistance response. Curr. Biol..

[117] Dubiella U., Seybold H., Durian G., Komander E., Lassig R., Witte C.P., Schulze W.X., Romeis T. (2013). Calcium-dependent protein kinase/NADPH oxidase activation circuit is required for rapid defense signal propagation. Proc. Natl. Acad. Sci. USA.

[118] Torres M.A., Dangl J.L., Jones J.D.G. (2002). *Arabidopsis* gp91phox homologues, AtrbohD and AtrbohF are required for accumulation of reactive oxygen intermediates in the plant defense response. Proc. Natl. Acad. Sci. USA.

[119] Moeder W., Urquhart W., Ung H., Yoshioka K. (2011). The role of cyclic nucleotide-gated ion channels in plant immunity. Mol. Plant.

[120] Shih H.W., DePew C.L., Miller N.D., Monshausen G.B. (2015). The cyclic nucleotide-gated channel CNGC14 regulates root gravitropism in *Arabidopsis thaliana*. Curr. Biol..

[121] Nguyen H.T., Umemura K., Kawano T. (2016). Indole-3-acetic acid-induced oxidative burst and an increase in cytosolic calcium ion concentration in rice suspension culture. Biosci. Biotechnol. Biochem..

[122] Peer W.A., Cheng Y., Murphy A.S. (2013). Evidence of oxidative attenuation of auxin signalling. J. Exp. Bot..

[123] Maruta T., Inoue T., Tamoi M., Yabuta Y., Yoshimura K., Ishikawa T., Shigeoka S. (2011). *Arabidopsis* NADPH oxidases, AtrbohD and AtrbohF, are essential for jasmonic acid-induced expression of genes regulated by MYC2 transcription factor. Plant Sci..

[124] Lu M., Zhang Y., Tang S., Pan J., Yu Y., Han J., Li Y., Du X., Nan Z., Sun Q. (2016). AtCNGC2 is involved in jasmonic acid-induced calcium mobilization. J. Exp. Bot..

[125] Kawano T., Sahashi N., Takahashi K., Uozumi N., Muto S. (1998). Salicylic acid induces extracellular superoxide generation followed by an increase in cytosolic calcium ion in tobacco suspension culture: The earliest events in salicylic acid signal transduction. Plant Cell Physiol..

[126] Manzoor H., Kelloniemi J., Chiltz A., Wendehenne D., Pugin A., Poinssot B., Garcia-Brugger A. (2013). Involvement of the glutamate receptor AtGLR3.3 in plant defense signaling and resistance to *Hyaloperonospora arabidopsidis*. Plant J..

[127] Jayakannan M., Bose J., Babourina O., Rengel Z., Shabala S. (2015). Salicylic acid in plant salinity stress signalling and tolerance. Plant Growth Regul..

[128] Wang Y.F., Munemasa S., Nishimura N., Ren H.M., Robert N., Han M., Puzõrjova I., Kollist H., Lee S., Mori I., Schroeder J.I. (2013). Identification of cyclic GMP-activated nonselective Ca^2+^-permeable cation channels and associated CNGC5 and CNGC6 genes in *Arabidopsis* guard cells. Plant Physiol..

[129] Gémes K., Kim Y.J., Park K.Y., Moschou P.N., Andronis E., Valassaki C., Roussis A., Roubelakis-Angelakis K.A. (2016). An NADPH-oxidase/polyamine oxidase feedback loop controls oxidative burst under salinity. Plant Physiol..

[130] Guo K.M., Babourina O., Christopher D.A., Borsics T., Rengel Z. (2008). The cyclic nucleotide-gated channel, AtCNGC10, influences salt tolerance in *Arabidopsis*. Physiol. Plant.

[131] Guo K.M., Babourina O., Christopher D.A., Borsics T., Rengel Z. (2010). The cyclic nucleotide-gated channel AtCNGC10 transports Ca^2+^ and Mg^2+^ in *Arabidopsis*. Physiol. Plant.

[132] Larkindale J., Hall J.D., Knight M.R., Vierling E. (2005). Heat stress phenotypes of *Arabidopsis* mutants implicate multiple signaling pathways in the acquisition of thermotolerance. Plant Physiol..

[133] Guo J., Zeng W., Chen Q., Lee C., Chen L., Yang Y., Cang C., Ren D., Jiang Y. (2016). Structure of the voltage-gated two-pore channel TPC1 from *Arabidopsis thaliana*. Nature.

